# Increased Risk of Respiratory Mortality Associated with the High-Tech Manufacturing Industry: A 26-Year Study

**DOI:** 10.3390/ijerph13060557

**Published:** 2016-06-03

**Authors:** Ro-Ting Lin, David C. Christiani, Ichiro Kawachi, Ta-Chien Chan, Po-Huang Chiang, Chang-Chuan Chan

**Affiliations:** 1 Takemi Program in International Health, Department of Global Health and Population, Harvard T.H. Chan School of Public Health, Boston, MA 02115, USA; 2 Department of Environmental Health, Harvard T.H. Chan School of Public Health, Boston, MA 02115, USA; dchris@hsph.harvard.edu; 3 Department of Epidemiology, Harvard T.H. Chan School of Public Health, Boston, MA 02115, USA; 4 Department of Social and Behavioral Sciences, Harvard T.H. Chan School of Public Health, Boston, MA 02115, USA; ikawachi@hsph.harvard.edu; 5 Research Center for Humanities and Social Sciences, Academia Sinica, Taipei 11529, Taiwan; tachien@gate.sinica.edu.tw; 6 Institute of Population Health Sciences, National Health Research Institutes, Miaoli County 35053, Taiwan; chiangp@nhri.org.tw; 7 Institute of Occupational Medicine and Industrial Hygiene, College of Public Health, National Taiwan University, Taipei 10055, Taiwan; ccchan@ntu.edu.tw; 8 Global Health Center, College of Public Health, National Taiwan University, Taipei 10055, Taiwan

**Keywords:** environment and public health, economic development, industrial development, socioeconomic factors, asthma, chronic obstructive pulmonary disease

## Abstract

Global high-tech manufacturers are mainly located in newly industrialized countries, raising concerns about adverse health consequences from industrial pollution for people living nearby. We investigated the ecological association between respiratory mortality and the development of Taiwan’s high-tech manufacturing, taking into account industrialization and socioeconomic development, for 19 cities and counties—6 in the science park group and 13 in the control group—from 1982 to 2007. We applied a linear mixed-effects model to analyze how science park development over time is associated with age-adjusted and sex-specific mortality rates for asthma and chronic obstructive pulmonary disease (COPD). Asthma and female COPD mortality rates decreased in both groups, but they decreased 9%–16% slower in the science park group. Male COPD mortality rates increased in both groups, but the rate increased 10% faster in the science park group. Science park development over time was a significant predictor of death from asthma (*p* ≤ 0.0001) and COPD (*p* = 0.0212). The long-term development of clustered high-tech manufacturing may negatively affect nearby populations, constraining health advantages that were anticipated, given overall progress in living standards, knowledge, and health services. National governments should incorporate the long-term health effects on local populations into environmental impact assessments.

## 1. Introduction

**Figure 1:**
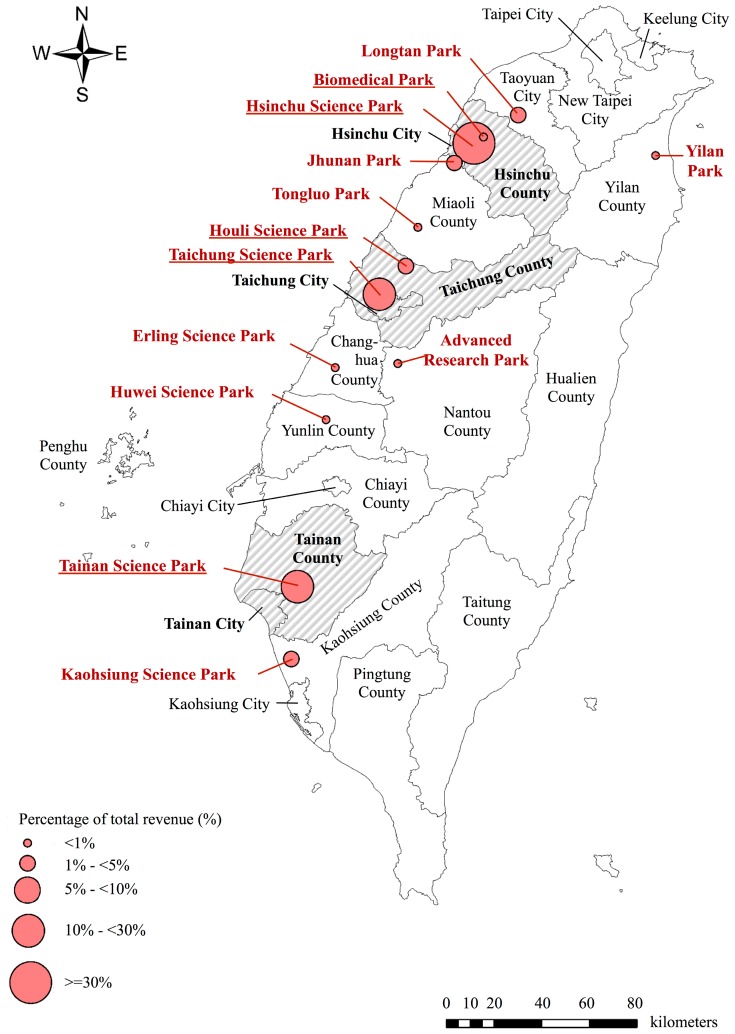
Science park locations in Taiwan, with percentage of total science park revenue, 2015. Circles are drawn in proportion to the percentage of the local science park’s revenue compared with total science park revenue in Taiwan in 2015 [1].

Globalization is reshaping the high-tech global supply chain. Many high-tech manufacturers have relocated to newly industrialized countries. Increasing global demand for mobile devices and access to the Internet has propelled the growth of Taiwan’s high-tech industries. The Global Competitiveness Report 2014–2015 ranked Taiwan second in the “state of cluster development” index, indicating that the country has well-developed and concentrated clusters of firms, suppliers, producers, and specialized industrial institutions. As of 2015, Taiwan had 13 designated science parks (Figure 1). Of these 13 science parks, 90% of science park economic activity was attributed to activities in six cities and counties (Table 1) [1]. Companies in these science parks were clustered mainly in the high-tech manufacturing industry, highly important to the global supply chain [2].

Taiwan’s high-tech industrial clusters have been key drivers of the country’s successful economic growth over the past 30 years, increasing economic activity, generating employment, attracting highly educated people, and forming urban settings near the industrial areas. In 2015, Taiwan’s science parks created 2.31 trillion in revenue in New Taiwan Dollars (NTD) (72 billion in United States Dollars (USD), based on an average exchange rate of 1 NTD = 0.0313 USD in 2015), making up 14% of the country’s gross domestic product (GDP) [1,3]. These science parks have provided an average per month of 264,777 jobs, amounting to 2.36% of total employees in Taiwan in 2015 [1].

The development of industrial clusters can change a country’s industrial and labor structure, as well as affect environmental quality and population health. Many studies have monitored environmental quality near the high-tech manufacturing industry in Taiwan and identified several ambient air pollutants—such as volatile organic compounds (VOCs) as well as acid and base air pollutants—associated with emissions [4,5,6,7]. Even though the releases of specific air pollutants have been identified, health effects from these pollutants have not been determined. Taiwan’s first high-tech manufacturing science park was developed in 1980—12 years after the establishment of the first petrochemical industry in 1968. Previous studies of petrochemical industrialization have found increased respiratory symptoms, increased cancer mortality, and shortened life expectancy in communities near high-polluting petrochemical areas [8,9,10]. However, environmental exposure and associated health issues have been reported rarely for areas near Taiwan’s high-tech manufacturing science parks [11].

The existence of environmental quality standards does not guarantee complete health protection from industrial pollution. Chemicals consumed in the high-tech manufacturing industry often outpace environmental standards [12]. The Environmental Impact Assessment (EIA) Act of Taiwan, promulgated in 1994, is meant to protect population health before approving new expansions. Health impact assessment (HIA) is not yet legally required in all environmental impact reports under EIA Act in Taiwan. However, for some projects that were asked to perform HIA, some researchers and nongovernmental organizations have criticized its current practice for two reasons. First, the Taiwan HIA was influenced by political power, and the final decision-making process lacked information transparency and public participation [13,14]. Second, the HIA’s common practice of assessing “only additional risk associated with the new expansion of a project site” instead of “total health risk associated with the whole science park area” has also prompted criticism [15].

Following increased public awareness of environmental protection and subsequent concerns about adverse health consequences from industrial pollution, local farmers and environmental activists have protested against further expansion of high-tech manufacturing science parks [15]. An epidemiological analysis of how health consequences are related to industrial development can provide justification for decision-makers who are considering whether to develop new industrial areas or not. Publicly available city- and county-level data offer an opportunity to address these important issues through data analysis. The objective of this study was to investigate the ecological association between science park development and respiratory mortality, taking into account industrialization and socioeconomic development. We hypothesized that there is a substantial adverse health effect associated with industrial development over time.

## 2. Materials and Methods

### 2.1. Data: Study Period and Geographic Units

We used two criteria to choose the study period: (1) consistency and availability of city- or county-level data; and (2) consistency of definitions of outcome indicators, to improve data comparability. First, according to the International Organization for Standardization (ISO) codes for the names of the principle subdivisions of Taiwan, *i.e.*, ISO 3166-2:TW, Taiwan had 23 cities and counties during the period 1982–2009, including two special municipalities (here called cities), five municipalities (cities), and 16 districts (counties). Second, Taiwan adopted the International Classification of Diseases, ninth revision (ICD-9), during the period 1979–2007, then implemented ICD-10 in 2008. On the basis of criteria (1) and (2), we selected 1982–2007 as our study period. Among the 23 cities and counties, we excluded one outlying island and three petrochemical manufacturing cities and counties (Table 2). Our final analysis included 19 cities and counties, which represented 84.14% of Taiwan’s population in 2015. We treated the 6 cities and counties with high-tech manufacturing science parks nearby as the science park group and the other 13 cities and counties as the control group.

### 2.2. Data: Dependent Variables

The measure used to indicate city or county population health status was respiratory mortality. The respiratory diseases studied were asthma (ICD-9 Code: 493) and chronic obstructive pulmonary disease (COPD; ICD-9 Codes: 490–492, 494, and 496). For each city or county, the sex-specific mortality rate for each disease was calculated and then age-standardized to the World Standard Population published by the World Health Organization in 2000. Mortality and demographic data covering the period 1982–2007 were obtained from the Ministry of Health and Welfare and the Ministry of Interior of Taiwan, respectively [16,17].

### 2.3. Data: Independent Variables

We identified nine potential ecological determinants of respiratory mortality (Table 3). We treated the designation of an area for science park development over time as our primary exposure of interest. We adjusted for four indicators of industrialization: unemployment rate (measured as percentage); revenue of registered manufacturing factories (measured in trillion NTD); particulate matter (measured in 10 μg/m^3^); and traffic density (measured by 1000 cars/motorcycles per km^2^) [18,19,20]. We also examined four indicators of socioeconomic development: education (measured as percentage completing senior high school); medical services (measured as number of registered medical professionals per hundred population); household disposable income (measured in million NTD); and relative poverty (measured as percentage of households with disposable income below 50% of the median equivalized income) [19,20,21]. For missing observations, we used a natural spline to interpolate time-related dependency datasets.

### 2.4. Data Analysis

Data from the science park group were compared with data from the control group during the period from 1982 to 2007. The ecological associations between the selected determinants on yearly age-adjusted and sex-specific asthma and COPD mortality rates were analyzed using a linear mixed-effects model:

*log(Age-adjusted and sex-specific mortality rate)_i,j_ =* β*_0_ +* β*_1_ × (Group)_i,j_ +* β*_2_ × (Time)_i,j_ +* β*_3_ × (Group × Time)_i,j_ +* β*_4_ × (Unemployment)_i,j_ +* β*_5_ × (Manufacturing revenue)_i,j_ +* β*_6_ × (Particulate matter)_i,j_ +* β*_7_ × (Traffic density)_i,j_ +* β*_8_ × (Education)_i,j_ +* β*_9_ × (Medical services)_i,j_ +* β*_10_ × (Household income)_i,j_ +* β*_11_ × (Relative poverty)_i,j_ +* β*_12_ × (Sex)_i,j_ + b_0i_ + b_1i_ × (Time)_i,j_ + b_2i_ × (Unemployment)_i,j_ + b_3i_ × (Manufacturing revenue)_i,j_ + b_4i_ × (Particulate matter)_i,j_ + b_5i_ × (Traffic density)_i,j_ + b_6i_ × (Education)_i,j_ + b_7i_ × (Medical services)_i,j_ + b_8i_ × (Household income)_i,j_ + b_9i_ × (Relative poverty)_i,j_ + b_10i_ × (Sex)_i,j_ +* ε*_i,j_* ,

where β denotes coefficient estimates for the fixed effects, *b* denotes coefficient estimates for the random effects, *i* ∈ (1, 2, …, 19) denotes the city or county code, *j* ∈ (1, 2, …, 26) denotes the calendar year from 1982 to 2007, and ε denotes the error term. Cities and counties of the science park group were sequentially added into analysis at the point that science parks were established. All statistical analyses were performed using SAS version 9.3 (SAS Institute, Cary, NC, USA).

## 3. Results

Table 4 shows the averaged means for asthma and COPD mortality rates, the four industrialization determinants, and the four socioeconomic determinants. The science park group is compared with the control group. To calculate the averaged means for each indicator, we divided the years into four periods: 1982–1987, 1988–1994, 1995–2001, and 2002–2007. Originally, in the periods 1982–1987 and 1988–1994, the science park group included only Hsinchu City and Hsinchu County. Following the establishment of South Taiwan Science Park in 1997, Tainan City and Tainan County were added to the science park group (*n* = 4 in 1995–2001). Finally, Taichung City and Taichung County were added (*n* = 6 in 2002–2007), following the establishment of Central Taiwan Science Park in 2003.

During all four time periods, asthma and COPD mortality rates were lower in the science park group than in the control group; however, as time went by, the difference between two groups became smaller. The asthma mortality rates decreased in both groups, but they decreased 10%–16% more slowly in the science park group (Table 4). Male COPD mortality rates increased in both groups, but the mortality rate increased 10% faster in the science park group. The female COPD mortality rates decreased in both groups, but the mortality rate decreased 9% more slowly in the science park group. During these same periods, the annual rates of change in manufacturing revenue and traffic density were 57% and 19% higher in the science park group. For the other determinants—unemployment, particulate matter, education, medical services, household income, and relative poverty—the differences between the science park group and the control group were less than 8%.

Table 5 shows the modeling results for the selected determinants of respiratory mortality. The asthma mortality rate was lower in the science park group than in the control group (*p* = 0.0020). Even though overall asthma mortality decreased over time (*p* = 0.0008), it decreased at a slower speed in the science park group compared with the control group (*p* ≤ 0.0001). Similarly, the COPD mortality rate was lower in the science park group than in the control group (*p* = 0.0247). As time went by, the COPD mortality rate increased at a faster rate in the science park group than in the control group (*p* = 0.0212). For the effects of other selected determinants, asthma mortality was inversely associated with education (*p* = 0.0190) and medical services (*p* = 0.0174), and positively associated with household income (*p* = 0.0003). COPD mortality was positively associated with unemployment (*p* = 0.0005) and inversely associated with education (*p* = 0.0004).

## 4. Discussion

### 4.1. Main Findings

The development of high-tech industrial areas may result in a lack of increased health advantages for nearby populations, contrary to what would be expected, given overall improvements in living standards, knowledge, and health services. The longer the science parks existed, the slower the declines observed in rates of asthma and female COPD mortality. Male COPD mortality rates actually increased at a faster speed in the science park group.

### 4.2. Possible Explanation

Air pollutants associated with the high-tech manufacturing emissions are the major health concern of science park development [4,5,6,7]. Exposure not only to particulate matter but also to several ambient air pollutants, such as sulfur oxides, nitrogen oxides, ozone, and VOCs, may increase the risk of respiratory diseases [22]. In the short term, when the concentration of each sulfur dioxides, nitrogen dioxides, and ozone over 10 μg/m^3^, the excess cases of hospital admissions for COPD increased by 0.5%, 0.7%, and 2%, respectively [23]. In the long term, chronic exposure to air pollutants not only increases the risk of asthma among children but also contributes to the development of asthma-COPD overlap syndrome (ACOS) [24,25,26]. Residents working in high-tech manufacturing factories may also be exposed to toxic chemicals at their workplaces [27]. If the environmental quality near industrial clusters fails to improve at a faster speed, emissions of air pollutants from industrial clusters might be harmful to the health of people living nearby in the long run.

Fast-paced and high-pressure environments are common working conditions in the high-tech industry. According to a previous nationwide study in Taiwan, anxiety is another risk factor for asthma [28]. Exposure to stress and other psychological factors might aggravate pre-existing or induce new-onset asthma among residents who work in the high-tech industry. For these workers, reducing workplace stress through better workplace conditions or environments as well as adjustments in job design might result in health improvements [29,30].

Since the science park development functions as a surrogate indicator of industrial air pollution and high-pressure environments, government should target improving environmental quality and working conditions to improve the overall health of workers and residents.

Higher socioeconomic development should be associated with lower rates of respiratory diseases [31,32]. However, our findings that household income failed to contribute to reducing asthma mortality support the assertion that rapid economic growth did not necessarily bring better health outcomes [33]. Countries experiencing economic growth may also encounter accompanying health risk factors, such as income inequality, unhealthy urban environments, work-related stress, and air pollution. The net influence of the economy on health may in reality prove to be negative. Unless all these determinants can be improved together, along with rising living standards, the development of the high-tech manufacturing industry sector will be a mixed blessing for local populations.

### 4.3. Study Limitations and Strengths

Several limitations should be noted. First, our findings should be interpreted at the city and county level, within the constraints of the ecological design. Differing extents of high-tech industrial development may have different impacts on subareas, subgroups, and individuals—as such, a separate analysis is warranted to differentiate exposure and healthy worker effects. The collection and use of data that reflect personal socioeconomic level and/or detailed geographic boundaries might provide more accurate estimations in future studies of the impact of science park development.

Second, smoking is also a risk factor for respiratory diseases, and previous literature has observed elevated smoking prevalence, sometimes double, among people with lower socioeconomic status [34,35]. Socioeconomic determinants were regarded as surrogates of smoking in our study. We assumed that the group with higher education and higher income had lower smoking prevalence in our study. Because Taiwan’s high-tech industry absorbed highly educated and skilled manpower—27% of science park workers held a graduate-level education in 2015 [1]—our estimation of the negative health effects of science parks is unlikely to be an overestimation.

A third limitation might be the possible adverse health effects of migration—if people were moving from areas with higher asthma prevalence to high-tech industrial areas that originally had lower rates of respiratory diseases, this might contribute to the finding of increased mortality. However, this concern may be insubstantial given the plausible healthy worker effect and the effect of the hosting environment. Workers—who in this scenario are those who migrate for jobs in manufacturing factories—usually have lower mortality rates than the general population. Those who are severely ill and chronically disabled are often excluded from employment. In addition, previous literature has indicated that asthma prevalence in immigrants converges toward the prevalence in the hosting environment [36]. Thus, our finding of the adverse health effects of science parks is less likely to be overestimated.

Despite these limitations, our longitudinal analysis represents an improvement on current health risk assessment in Taiwan. First, this study’s strength was its analysis of 26 years of data (1982 to 2007) that encompass three stages of science park development, from establishment to young, middle, and mature. Second, we took into account the extent of socioeconomic development during the same period. We could, therefore, determine that the length of time since the science park establishment is relevant to the adverse effect on health.

## 5. Conclusions

Globalization has increased the number of high-tech manufacturing centers in newly industrialized countries and the associated risks to population health. The growing public awareness of environmental protection and community health has made the decision to expand industrial areas much more difficult now than several years ago. These decisions are particularly important to governments such as Taiwan’s and of other newly industrialized countries that pursue economic growth.

Our findings highlight the long-term adverse impact of science parks on population health in Taiwan. National governments should consider strengthening environmental impact assessments, which should (1) incorporate the long-term health effects in the process of environmental impact assessments; (2) continuously monitor health impacts over the long term; and (3) assess the impact of industrial cluster as a whole instead of limiting it to incremental health risks associated solely with the new expansion areas. Global businesses should consider disclosure of the impact of their suppliers’ activities on local environments and community health as a critical component of sound corporate social responsibility.

## Figures and Tables

**Table 1:** Science parks in Taiwan.

Science Parks	Date Established	Land Occupancy Rate ^1^	Major Industries ^2^	Distance ^3^
Hsinchu Science Park (HSP): (1) Hsinchu Science Park, (2) Jhunan Science Park, (3) Longtan Science Park, (4) Biomedical Park, (5) Tongluo Science Park, (6) Yilan Science Park	15 December 1980	85.74%	Integrated circuits, Optoelectronics	5–6 km
South Taiwan Science Park (STSP): (1) Tainan Science Park, (2) Kaohsiung Science Park	8 July 1997	89.69%	Integrated circuits, Optoelectronics	17–18 km
Central Taiwan Science Park (CTSP): (1) Taichung Science Park, (2) Huwei Science Park, (3) Houli Science Park, (4) Erling Science Park, (5) Advanced Research Park	21 March 2003	90.36%	Integrated circuits, Optoelectronics	12–13 km

**^1^** Calculated by dividing occupied rentable area by total rentable area in December 2015 [1]. **^2^** Listed industry with turnover accounted for more than 5% of each science park in 2015 (see Supplementary Table S1). **^3^** Distance from the science park bureau to the nearest city’s central train station.

**Table 2:** Cities and counties in each group and nearby science parks, economic activity, and population.

City and County (by Alphabetical Order)	Nearby Science Parks	Percentage of Science Park Economic Activity in 2015	Population (%) in December 2015 ^1^
**Science Park Group**			
Hsinchu City and Hsinchu County **^2^**	Hsinchu Science Park and Biomedical Park	41.52%	976,102 (4.16%)
Taichung City and Taichung County **^3^**	Taichung Science Park and Houli Science Park	20.87%	2,744,445 (11.68%)
Tainan City and Tainan County **^4^**	Tainan Science Park	28.79%	1,885,541 (8.03%)
**Control Group**			
Changhua County	Erling Science Park	0.03%	1,289,072 (5.49%)
Chiayi City	-	-	270,366 (1.15%)
Chiayi County	-	-	519,839 (2.21%)
Hualien County	-	-	331,945 (1.41%)
Keelung City	-	-	372,105 (1.58%)
Miaoli County	Jhunan Park and Tongluo Park	3.29%	563,912 (2.40%)
Nantou County	Advanced Research Park	0.01%	509,490 (2.17%)
New Taipei City	-	-	3,970,644 (16.90%)
Pingtung County	-	-	841,253 (3.58%)
Taipei City	-	-	2,704,810 (11.51%)
Taitung County	-	-	222,452 (0.95%)
Taoyuan City	Longtan Park	2.90%	2,105,780 (8.96%)
Yilan County	Yilan Park	-	458,117 (1.95%)
**Exclusion Group**			
Kaohsiung City and Kaohsiung County **^5^**	Kaohsiung Science Park	2.19%	2,778,918 (11.83%)
Penghu County **^6^**	-	-	102,304 (0.44%)
Yunlin County **^5^**	Huwei Science Park	0.41%	699,633 (2.98%)

**^1^** Total population was 23,492,074, including Kinmen County and Lienchiang County, which were added into ISO 3166-2: TW in 2015. **^2^** The Hsinchu Science Park covers Hsinchu City and Hsinchu County geographically. The economic activity was reported together. **^3^** Taichung City and Taichung County were merged into one municipality in 2010. The population was reported together in 2015. **^4^** Tainan City and Tainan County were merged into one municipality in 2010. The population was reported together in 2015. **^5^** Petrochemical manufacturing areas. **^6^** Outlying island.

**Table 3:** Definition of the selected determinants of health.

Term	Definition
Science park establishment	Whether or not the science park was established at the point of study year (Table 1)
**Industrialization**	
Unemployment	Percentage of employable people in a city’s or county’s workforce who are 15 years of age or older and either actively seeking jobs or willing to work anytime
Manufacturing revenue	Annual total revenue of registered factories in manufacturing industries, measured in trillion New Taiwan Dollar
Particulate matter	Annual averaged concentrations of total ambient particulate matter, measured in 10 μg/m^3^
Traffic density	Number of cars and motorcycles, measured in 1000 per km^2^
**Socioeconomic Development**	
Education	Percentage of the population 15 years of age or older who completed senior high school
Medical services	Number of registered medical personnel per hundred population
Household income	Averaged disposable income per household **^1^**, measured in million New Taiwan Dollar
Relative poverty	Percentage of households with disposable income levels below 50% of the median equivalized income **^2^**

**^1^** Adjusted for Consumer Price Index. **^2^** Relative poverty was defined as the percentage of households with disposable income levels below 50% of the median equivalized income of each city or county. The equivalized income was created by adjusting for extreme values (*i.e.*, income less than zero was treated as zero, and income above ten times the median was treated as ten times median).

**Table 4:** Averaged means and standard deviations (SDs) for city- and county-level respiratory mortality rate and its determinants, 1982–2007.

Determinant ^1^	Science Park Group ^2^ Mean (SD)						Control Group Mean (SD)				
	1982–1987 *n* = 2	1988–1994 *n* = 2	1995–2001 *n* = 4	2002–2007 *n* = 6	Average Change ^3^		1982–1987 *n* = 13	1988–1994 *n* = 13	1995–2001 *n* = 13	2002–2007 *n* = 13	Average Change ^3^
Asthma mortality **^4^**	7.72 (2.70)	7.81 (2.06)	6.41 (2.35)	3.68 (1.28)	−19.76%		12.47 (5.11)	9.47 (3.99)	6.99 (3.35)	4.06 (1.86)	−30.72%
Males	9.75 (3.52)	10.11 (3.21)	7.65 (3.04)	4.26 (1.71)	−21.66%		15.10 (6.23)	11.66 (4.86)	8.16 (3.72)	4.80 (2.07)	−31.31%
Females	5.19 (2.91)	5.16 (2.11)	5.08 (2.06)	3.10 (1.35)	−13.67%		9.94 (5.05)	7.44 (3.91)	5.80 (3.51)	3.29 (1.90)	−30.12%
COPD mortality **^4^**	12.29 (3.34)	10.59 (1.88)	16.07 (3.02)	14.37 (3.33)	9.10%		18.06 (4.94)	15.77 (5.65)	19.68 (6.15)	17.49 (5.26)	0.33%
Males	15.98 (5.63)	15.37 (3.63)	23.69 (5.61)	22.32 (5.65)	14.83%		23.58 (6.15)	22.05 (7.52)	28.23 (9.11)	26.12 (7.72)	4.69%
Females	8.28 (4.13)	5.66 (2.44)	8.54 (2.04)	6.68 (2.08)	−0.83%		13.21 (5.58)	9.75 (4.68)	11.21 (4.51)	9.22 (4.11)	−9.65%
Unemployment	2.26 (0.77)	1.47 (0.53)	2.90 (1.10)	4.33 (0.52)	37.14%		2.56 (1.13)	1.69 (0.65)	2.92 (0.95)	4.45 (0.54)	30.40%
Manufacturing revenue **^5^**	0.07 (0.03)	0.15 (0.07)	0.41 (0.22)	0.63 (0.34)	115.57%		0.12 (0.17)	0.20 (0.30)	0.36 (0.58)	0.46 (0.76)	58.59%
Particulate matter	11.22 (4.21)	10.37 (2.10)	10.45 (2.01)	8.52 (1.34)	−8.44%		13.35 (3.58)	12.34 (4.32)	9.50 (2.87)	7.92 (1.73)	−15.74%
Traffic density	0.66 (0.60)	1.12 (1.03)	1.67 (1.42)	2.37 (2.03)	53.70%		0.57 (0.87)	0.90 (1.36)	1.15 (1.63)	1.36 (1.86)	35.00%
Education	38.02 (3.53)	46.33 (3.57)	54.52 (6.21)	64.71 (8.13)	19.41%		35.56 (8.80)	43.07 (9.37)	49.94 (10.16)	57.42 (11.60)	17.35%
Medical services	0.29 (0.12)	0.37 (0.13)	0.59 (0.15)	0.88 (0.29)	46.23%		0.30 (0.15)	0.44 (0.20)	0.65 (0.29)	0.87 (0.32)	42.90%
Household income **^5^**	0.53 (0.08)	0.82 (0.11)	0.98 (0.14)	0.98 (0.16)	24.66%		0.48 (0.08)	0.74 (0.17)	0.91 (0.16)	0.88 (0.17)	25.30%
Relative poverty	9.87 (2.44)	13.26 (3.53)	15.46 (5.25)	14.26 (5.96)	14.41%		12.29 (6.20)	16.71 (9.05)	18.84 (9.30)	21.29 (9.81)	20.57%

*n* = number of cities and counties; *COPD* = chronic obstructive pulmonary disease; NTD = New Taiwan Dollar; **^1^** definitions of the determinants of health are shown in Table 3; **^2^** based on the year that a science park was established in the city or county; **^3^** calculated by averaging the differences between two periods divided by the previous period; **^4^** per 100,000 population; **^5^** one NTD = 0.0263 United States Dollar between 1982 and 1987, 0.0375 between 1988 and 1994, 0.0328 between 1995 and 2001, 0.0300 between 2002 and 2007 [3].

**Table 5:** Estimated fixed effects of determinants on city- and county-level respiratory mortality rates.

Determinant ^1^	Asthma ^2^			COPD ^2^		
	Estimate	SE	*p* Value	Estimate	SE	*p* Value
**Intercept**	2.6703	0.2946	<0.0001	2.9940	0.2472	<0.0001
**Science Park Development**						
Science park establishment (yes *vs.* no)	−0.5632	0.1522	0.0002	−0.2871	0.1275	0.0247
Time (years since 1982)	−0.0400	0.0099	0.0008	0.0165	0.0087	0.0749
Science park establishment × Time	0.0324	0.0080	<0.0001	0.0132	0.0057	0.0212
**Industrialization**						
Unemployment	0.0073	0.0191	0.7062	0.0673	0.0159	0.0005
Manufacturing revenue	−0.1823	0.1429	0.2184	0.0526	0.0726	0.4785
Particulate matter	0.0040	0.0061	0.5138	0.0044	0.0045	0.3385
Traffic density	0.0734	0.0921	0.4352	0.0226	0.0879	0.7995
**Socioeconomic Development**						
Education	−0.0198	0.0076	0.0190	−0.0289	0.0065	0.0004
Medical services	−0.5139	0.1938	0.0174	0.0082	0.1911	0.9665
Household income	0.7249	0.1592	0.0003	0.1875	0.1385	0.1947
Relative poverty	0.0066	0.0042	0.1396	−0.0025	0.0033	0.4605
Sex (male *vs.* female)	0.4219	0.0407	<0.0001	0.9488	0.0441	<0.0001

COPD = chronic obstructive pulmonary disease; SE = standard error; **^1^** definitions of the determinants of health are shown in Table 3; **^2^** log-transformed age-adjusted mortality rate.

## Supplementary Materials

The following are available online at www.mdpi.com/1660-4601/13/6/557/s1, Table S1 Industries in each science park and their turnovers in 2015.

## Abbreviations

The following abbreviations are used in this manuscript:

COPD: chronic obstructive pulmonary disease
NTD: New Taiwan Dollars
USD: United States Dollars
GDP: gross domestic product
VOCs: volatile organic compounds
EIA: Environmental Impact Assessment
HIA: Health impact assessment
ISO: International Organization for Standardization
ICD: International Classification of Diseases
ACOS: asthma-COPD overlap syndrome
