# Supplementary material for: Increased Risk of Respiratory Mortality Associated with the High-Tech Manufacturing Industry: A 26-Year Study
Source: Int J Environ Res Public Health. 2016 Jun 3;13(6):557. doi: 10.3390/ijerph13060557 (PMC4924014; doi:10.3390/ijerph13060557)
Supplement: Supplementary file 1 [file ijerph-13-00557-s001.pdf]

Supplementary Materials: Increased Risk of Respiratory Mortality Associated with the High-Tech Manufacturing Industry: A 26-Year Study

Ro-Ting Lin, David C. Christiani, Ichiro Kawachi, Ta-Chien Chan, Po-Huang Chiang, Chang-Chuan Chan

Table S1. Industries in each science park and their turnovers [1] in 2015 [1].

Science Parks	Integrated Circuits	Optoelectronics	Precision Machinery	Computers and Peripherals	Telecommunications	Biotechnology	Other	Total
**All**	**14,472.32**	**6700.61**	**819.94**	**420.88**	**381.36**	**191.43**	**98.36**	**23,084.90**
	**(62.69%)**	**(29.03%)**	**(3.55%)**	**(1.82%)**	**(1.65%)**	**(0.83%)**	**(0.43%)**	**(100.00%)**
**Hsinchu Science Park (HSP)**	**8009.38**	**1894.51**	**276.60**	**373.83**	**305.44**	**91.29**	**61.32**	**11,012.37**
	**(72.73%)**	**(17.20%)**	**(2.51%)**	**(3.39%)**	**(2.77%)**	**(0.83%)**	**(0.56%)**	**(100.00%)**
Hsinchu Science Park	7787.74	814.47	189.05	366.37	304.00	59.97	61.32	9582.93
	(81.27%)	(8.50%)	(1.97%)	(3.82%)	(3.17%)	(0.63%)	(0.64%)	(100.00%)
Jhunan Science Park	129.08	473.56	87.54	2.26	1.44	30.32	-	724.20
	(17.82%)	(65.39%)	(12.09%)	(0.31%)	(0.20%)	(4.19%)	-	(100.00%)
Longtan Science Park	63.26	606.48	-	-	-	-	-	669.74
	(9.45%)	(90.55%)	-	-	-	-	-	(100.00%)
Biomedical Park	-	-	-	-	-	1.00	-	1.00
	-	-	-	-	-	(100.00%)	-	(100.00%)
Tongluo Science Park	29.30	-	-	5.20	-	-	-	34.50
	(84.93%)	-	-	(15.07%)	-	-	-	(100.00%)
Yilan Science Park	-	-	-	-	-	-	-	-
	-	-	-	-	-	-	-	-
**Southern Taiwan Science Park (STSP)**	**3699.66**	**2952.72**	**307.70**	**16.06**	**74.82**	**76.15**	**24.26**	**7151.37**
	**(51.73%)**	**(41.29%)**	**(4.30%)**	**(0.22%)**	**(1.05%)**	**(1.06%)**	**(0.34%)**	**(100.00%)**
Tainan Science Park	3694.98	2599.97	196.71	4.43	74.82	68.21	7.52	6646.64
	(55.59%)	(39.12%)	(2.96%)	(0.07%)	(1.13%)	(1.03%)	(0.11%)	(100.00%)
Kaohsiung Science Park	4.69	352.75	110.99	11.63	-	7.94	16.73	504.73
	(0.93%)	(69.89%)	(21.99%)	(2.30%)	-	(1.57%)	(3.31%)	(100.00%)

Table S1. *Cont.*

Science Parks	Integrated Circuits	Optoelectronics	Precision Machinery	Computers and Peripherals	Telecommunications	Biotechnology	Other	Total
**Central Taiwan Science Park (CTSP)**	**2763.28**	**1853.39**	**235.65**	**30.99**	**1.10**	**23.99**	**12.78**	**4921.17**
	**(56.15%)**	**(37.66%)**	**(4.79%)**	**(0.63%)**	**(0.02%)**	**(0.49%)**	**(0.26%)**	**(100.00%)**
Taichung Science Park	2433.44	1412.01	168.82	11.39	1.10	15.05	11.04	4052.86
	(60.04%)	(34.84%)	(4.17%)	(0.28%)	(0.03%)	(0.37%)	(0.27%)	(100.00%)
Huwei Science Park	-	57.72	15.38	17.70	-	1.89	1.74	94.42
	-	(61.13%)	(16.29%)	(18.75%)	-	(2.00%)	(1.84%)	(100.00%)
Houli Science Park	329.84	383.66	51.44	-	-	-	-	764.94
	(43.12%)	(50.16%)	(6.72%)	-	-	-	-	(100.00%)
Erling Science Park	-	-	-	-	-	7.00	-	7.00
	-	-	-	-	-	(100.00%)	-	(100.00%)
Advanced Research Park	-	-	-	1.89	-	0.05	-	1.94
	-	-	-	(97.42%)	-	(2.58%)	-	(100.00%)

[1] Unit: hundred million in New Taiwan Dollars (NTD); one NTD = 0.0313 United States Dollars (USD) in 2015 [2].

References

1. Ministry of Science and Technology, Taiwan. Science Parks: Common Statistics Form. Available online: https://ap0512.most.gov.tw/WAS2/English/AsScienceParkE.aspx (accessed on 28 February 2016).
2. Central Bank of the Republic of China, Taiwan. New Taiwan Dollar Exchange Rates. Available online: http://www.pxweb.cbc.gov.tw/dialog/statfile1L.asp?lang=1&strList=L (accessed on 1 February 2016).

© 2016 by the authors; licensee MDPI, Basel, Switzerland. This article is an open access article distributed under the terms and conditions of the Creative Commons by Attribution (CC-BY) license (http://creativecommons.org/licenses/by/4.0/).
